# Media portrayal of illness-related medical crowdfunding: A content analysis of newspaper articles in the United States and Canada

**DOI:** 10.1371/journal.pone.0215805

**Published:** 2019-04-23

**Authors:** Blake Murdoch, Alessandro R. Marcon, Daniel Downie, Timothy Caulfield

**Affiliations:** Health Law Institute, Faculty of Law, University of Alberta, Edmonton, Alberta, Canada; University of Toronto, CANADA

## Abstract

**Background:**

Medical crowdfunding is a growing phenomenon, and newspapers are publishing on the topic. This research analyzed how illness-related crowdfunding and crowdfunding campaigns have recently been represented in newspapers that are popular in the United States and Canada.

**Methods:**

A sample of 336 articles about medical crowdfunding published during the two year time period from October 7, 2015 to October 6, 2017 was produced using a Factiva search of the English language newspapers with the largest Canadian and United States readership. A coding frame was developed for and applied to the sample to analyze content.

**Results:**

Articles portrayed crowdfunding campaigns positively (43.75%) and neutrally (47.92%), but rarely negatively (4.76%). Articles mostly mentioned the crowdfunding phenomenon with a neutral characterization (93.75%). Few (8.63%) articles mentioned ethical issues with the phenomenon of crowdfunding. Ailments most commonly precipitating the need for a campaign included cancer (49.11%) and rare disease (as stated by the article, 36.01%). Most articles (83.04%) note where donations and contributions can be made, and 59.23% included a hyperlink to an online crowdfunding campaign website. Some articles (26.49%) mentioned a specific monetary goal for the fundraising campaign. Of the 70 (20.83%) articles that indicated the treatment sought may be inefficacious, was unproven, was experimental or lacked regulatory approval, 56 (80.00%) noted where contributions can be made and 36 (51.43%) hyperlinked directly to an online crowdfunding campaign.

**Conclusions:**

Crowdfunding campaigns are portrayed positively much more often than negatively, many articles promote campaigns for unproven therapies, and links directly to crowdfunding campaign webpages are present in most articles. Overall, crowdfunding is often either implicitly or explicitly endorsed.

## Introduction

Over the past several years, the flow of funds through online crowdfunding has grown at an explosive rate.[1,2] This has coincided with the rapid growth of companies providing crowdfunding platforms. For example, GoFundMe was valued at approximately $600 million when it struck a venture capital deal in 2015,[3] and has since been expanding: in January 2017, the company acquired the platform CrowdRise,[3] and in April 2018 it acquired YouCaring.[4] Although major uses of crowdfunding include charity and entrepreneurship, personal campaigns relating to health concerns are common online,[5] with platforms specifically targeting this in their advertising.[6,7]

Crowdfunding is a pathway to granting individuals and families experiencing hardship an opportunity that they might otherwise not have, but it is also a complex, poorly understood phenomenon that has social, ethical and economic risks and benefits. Bioethical, economic and statistical research relating to crowdfunding is growing. Snyder, Sisler, Gonzales and others have published work detailing some of the concerns with medical crowdfunding, including the lack of clarity surrounding who benefits from campaigns, what factors determine how resources are distributed, how access to medical care is affected, and how privacy is affected.[8–14] Snyder, Vox, Caulfield and others have also considered how unproven therapies are marketed and legitimized through campaigns, finding that millions of dollars have been raised to use them.[15–17] Renwick et al considered economic risks of health-related crowdfunding, finding several, including “inefficient priority setting, financial risks, unclear regulatory frameworks, issues of accountability, transparency and due diligence, and risk of fraud and money laundering.”[18] Nonetheless, crowdfunding continues to grow.

Media portrayals of crowdfunding are underexplored in the existing literature. Popular newspapers have long been reporting human interest stories focused on individuals with health conditions,[19] and, not surprisingly, there are stories about crowdfunding and the efforts of individuals to raise funds through this online platform. Media outlets covering and linking to crowdfunding or public solicitation create additional attention for campaigns, potentially leading to higher donations. Publicized campaigns that are overwhelmingly successful can experience fund management problems.[20,21] Another concern with media coverage of crowdfunding is that there may be instances where unproven therapies are promoted through the publication of articles about patients crowdfunding for them.[17]

Campaigns that achieve mass exposure can generate greatly increased donations in comparison with other campaigns for individuals experiencing similar circumstances,[9] and this advantages individuals with “large social networks, a sympathetic story to tell, or contacts in the media.”[8,22,23] The ability to reach an audience of hundreds of thousands of people with a single article almost certainly contributes to stratification of the “haves” and “have-nots” of the crowdfunding world. The higher the article’s popularity, the greater the mutual benefit to both the crowdfunder and the publisher. A “popularity contest” effect can occur, whereby campaign creators attempt to create the most compelling, emotionally appealing narrative possible to attract funding.[24–27] Consequently, crowdfunding can generate a culture of extreme competitiveness.[25]

Decisions to contribute to crowdfunding campaigns are likely influenced by a complex set of social, economic and psychological factors.[8] Media representations of crowdfunding may influence public perceptions regarding these campaigns and, as a result, impact donation decisions. The goal of this study was to analyze how illness-related crowdfunding and crowdfunding campaigns have recently been represented in popular English language United States and Canadian newspapers. Such articles, and especially those that link directly to active online crowdfunding campaigns, may have broader ethical and public health implications due to the increased exposure. An analysis of these stories also sheds light on how crowdfunding, as a phenomenon, is portrayed in the public sphere.

Our research plan was to assess the tone of popular newspapers’ portrayal of illness-related crowdfunding and crowdfunding campaigns, delineate the basic characteristics of campaigns covered, determine how and how often ethical concerns with crowdfunding were discussed, note how often unproven therapies were sought via crowdfunding, and assess the extent of hyperlinking to donation websites and other practices that could influence campaign contributions.

## Methods

In order to create a sample of news articles for analysis, we undertook a systematic search on the Factiva database (https://www.dowjones.com/products/factiva/) for the two year time period of October 7, 2015 to October 6, 2017. As noted, interest in crowdfunding has been intensifying in recent years. This time frame allowed for the capture of a dataset that reflects the media discourse over this period. Factiva is a news source database owned by Dow Jones with almost 33,000 sources including most major North American newspapers,[28] in which search inquiries can be performed and corresponding text—in this case, articles—can be downloaded. Search terms were selected through an iterative process to capture the widest possible net of illness-related crowdfunding articles, while excluding the vast number of articles focused on other topics, such as crowdfunding for violent physical trauma or business-related crowdfunding. The final search terms included the following parameters: the presence of one of: “crowdfunding”, “crowdsourcing”, “gofundme”, “gofundme.com”, “youcaring”, “youcaring.com”, “justgiving”, “justgiving.com”, “fundrazr”, “fundrazr.com”, or “generosity.com”; AND the presence of one of “disease”, “illness” or “cancer”; AND the presence of one of “health”, “treatment*”, “transplant*”, “drug*”, or “medication*”; but NOT “crash”, “accident”, “stabbing”, “shooting” or “funeral”.

We limited the search to articles with a wide reach to United States and Canadian audiences. For United States publications, we included articles from newspapers with at least one of the following: a top 25 ranking in average total circulation as of 2013,[29] a top 25 ranking in local audience for print and online combined as of 2013,[30] or a top 25 ranking by digital traffic for January 2015.[31] The latter qualification resulted in the inclusion of DailyMail.co.uk, Telegraph.co.uk and Independent.co.uk despite being British publications, as they are three of the most read online sources in the United States. For example, DailyMail.co.uk had the third highest digital traffic of any newspaper in the US.[31] In Canada, the most up to date newspaper readership data is found on the recently established Vividata database.[32] For this study we included the 30 most popular (print and digital) English language newspapers in Canada by audience and reach, as reported by Vividata in their fourth quarter 2015 report, which was the latest data available at the time of study commencement.[32] Sources that were not searchable on Factiva were excluded.

The search was performed and 24 duplicate articles were excluded, leaving 498. From these, 162 were excluded as their main topics were unrelated to the core research topic of illness-related crowdfunding, e.g., business-related crowdfunding campaigns, natural disaster responses and institution-level non-profit fundraising efforts. The final data set of fully coded articles included 336. A coding frame for analyzing the content of the articles and converting it to numerical data for analysis was developed using both inductive and deductive methods. The questions in the coding frame were chosen in order to: (1) determine the main topic or focus of the article; (2) identify the tone of the article in relation to crowdfunding and the specific crowdfunding campaign; (3) determine what ethical issues, if any, were considered; (4) determine what type of person started the campaign(s) covered, and what illness the campaign(s) concerned, and; (5) answer other yes/no questions about the article, including what links or connections were made to specific campaigns. A draft frame was developed and applied to a sample of 17 articles (approximately 5% of the data set). After review by a second coder, the frame was altered to better reflect, and thus accurately identify, common themes or components of the articles. A revised coding frame was applied to a sample of 24 articles, interpretive reliability of which was confirmed by a second coder, resulting in further minor changes to the coding frame and subsequent finalization.

Assessment of the tone of portrayal of crowdfunding and crowdfunding campaigns was based on analysis of the presence of stated benefits and supportive statements versus stated issues and unsupportive statements. An article predominantly supportive or predominantly mentioning benefits was coded as positive. An article predominantly unsupportive or predominantly mentioning issues was coded as negative. Articles merely mentioning crowdfunding or a crowdfunding campaign in passing, and focusing on an illness or another related topic, were categorized as neutral. Articles were also coded “neutral” if both positive and negative representations were made and a definitive emphasis on either side could not be determined.

After finalizing the frame, a content analysis was then performed by a single coder who coded all 336 articles.[33] The contents of the coding frame can be identified in the table and figures presented in the Results section. A random sample of 55 articles representing over 10% of 498 articles assessed for main topic was reassessed by a second individual for intercoder reliability testing. Of these 55 articles, 37 articles had relevant main topics and were then fully recoded, representing over 10% of the final sample of 336. Inter-coder agreement was calculated using methods from Miles and Huberman that calculate agreement as total agreements/(total agreements + disagreements).[34] One question about the type of intervention or treatment being sought was removed due to low agreement. There was substantial agreement (>75%) for all remaining coding questions (see S1 Text Supplementary Materials). The binary Yes/No coding questions were also analyzed under Cohen’s Kappa.[35] Intercoder reliability was achieved with an average score of 0.7806 including a 0 score, and an average score of 0.8781 excluding the 0 score. See S1 Text Supplementary Materials for more information.

## Results

**Fig 1:**
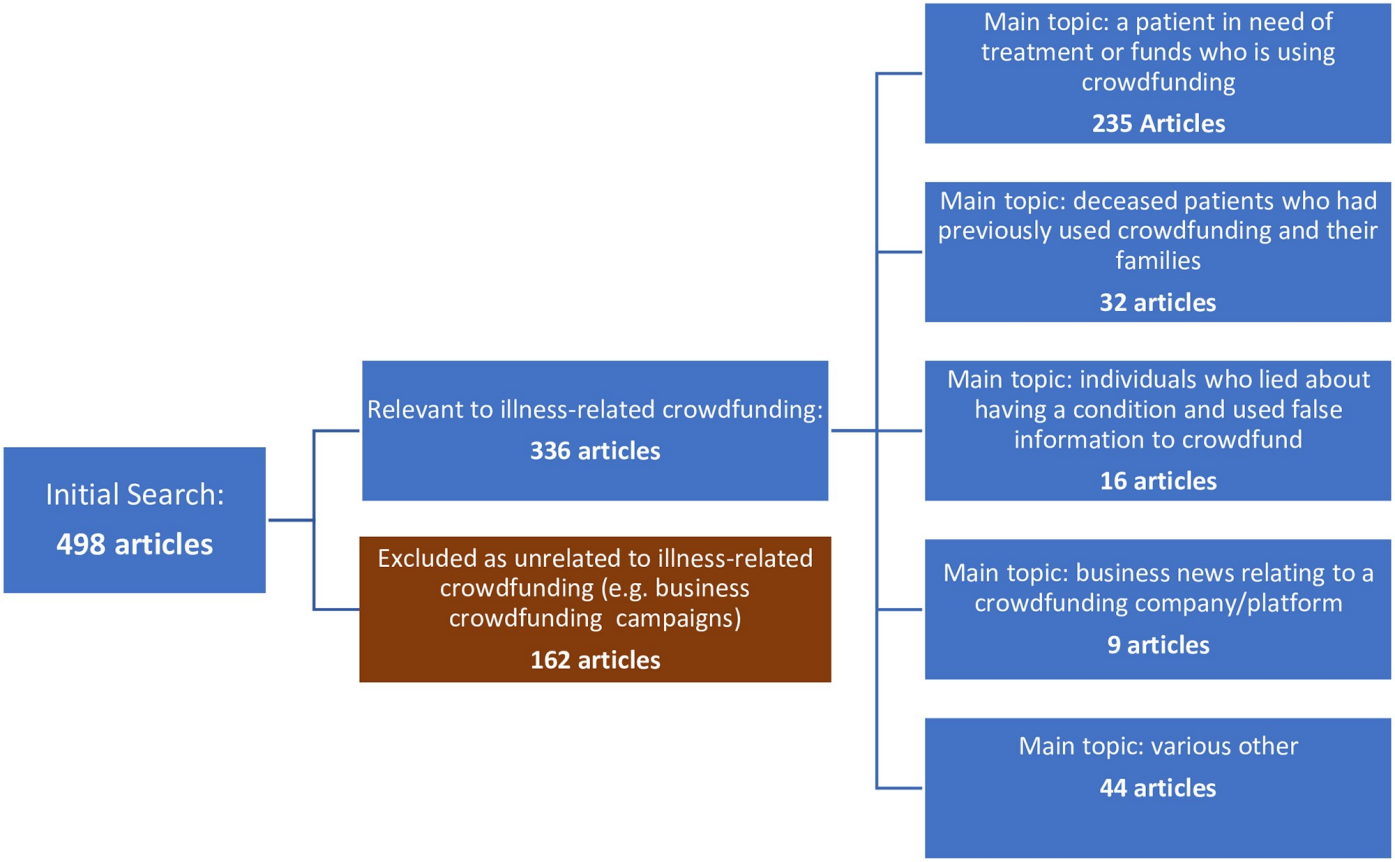
Sampling flow chart and breakdown of main topics.

See Fig 1 for a sampling flow chart and breakdown of articles’ main topics.

See Table 1 for the results for non-binary coding questions.

**Table 1:** Results from non-binary coding questions.

Coding Questions and Categories	Number of Responses	Percentage of Relevant Articles (336)
How is the phenomenon of crowdfunding portrayed?		
In a supportive or positive manner	7	2.08%
In a cautionary or negative manner	14	4.17%
With a neutral characterization	315	93.75%
Total	336	100.00%
If the article discusses a specific crowdfunding campaign, how is the campaign portrayed?		
In a positive or supportive manner	147	43.75%
In a negative or unsupportive manner	15	4.46%
In a neutral manner	161	47.92%
Does not cover a specific crowdfunding campaign	13	3.57%
Total	336	100.00%
If the article discusses ethical issues with the health-related crowdfunding phenomenon, what issues does it mention? Select all that apply.		
Crowdfunding for unproven therapies	6	1.79%
Privacy concerns	1	0.30%
Concerns over possible misuse of funds by campaign creator/manager or funding recipient	17	5.06%
Concern about equitable distribution of funds to those in need (i.e. donating to individuals instead of charity)	4	1.19%
Concern with fees and/or profits made by crowdfunding platforms	7	2.08%
Other (specify)	18	5.36%
Ethical issues with health-related crowdfunding were not discussed	307	91.37%
Total	360	-
If the article covers a specific patient or specific patients for whom crowdfunding is underway, who initiated the crowdfunding campaign? (choose all that apply)		
Patient	39	11.61%
Family	136	40.48%
Extended Family	1	0.30%
Friend(s)	35	10.42%
Stranger(s)	12	3.57%
Other (specify)	14	4.17%
No mention of who started the campaign	93	27.68%
No mention of specific campaign	13	3.87%
Total	343	-
If a specific crowdfunding campaign is covered, what ailment(s) is/are mentioned to have precipitated the need for a crowdfunding campaign?		
Cancer	165	49.11%
Rare Disease (as stated by the article)	121	36.01%
Organ Failure	3	0.89%
Other (Specify)	34	10.12%
A specific crowdfunding campaign is not covered	13	3.87%
Total	336	100.00%

The majority of articles (93.75%) mentioned the crowdfunding phenomenon (not the specific campaign) with a neutral characterization. However, there was a more even split between the characterization of specific campaigns, with similar numbers of articles portraying the subject campaign positively or supportively (43.75%) and neutrally (47.92%). Many articles focused on the condition or illness of a patient (patient was used in our coding frame to describe the subject of an illness-related crowdfunding campaign, regardless of whether that person’s treatment had yet begun). These articles usually mentioned crowdfunding in passing and often at the end, which constituted a neutral characterization but could still be viewed as an implicit endorsement. Others provided more details relating to a crowdfunding campaign, including statements from friends, family or the patient as to the positive effects of crowdfunding. There were very few articles (4.76%) that portrayed crowdfunding campaigns negatively, and those mostly concerned fraudulent behaviour.

Ethical issues with illness-related crowdfunding were sometimes discussed (8.63% of articles), most commonly in relation to the possible misuse of funds by the campaign creator or funding recipient (5.06%). This occurred mostly in articles about the fraudulent actors noted above. Fourteen of the 18 articles coded for “Other” raised ethical concerns about false stories being used to justify fraudulent crowdfunding.

Crowdfunding campaigns mentioned were initiated most commonly by the patient’s family (40.48%), though 27.68% of articles did not mention who started the campaign. Ailments most commonly mentioned as precipitating the need for a crowdfunding campaign included cancer (49.11%) and rare disease (as stated by the article, 36.01%). However, cancer was one of the search terms, so this impacted the representation of ailments mentioned.

**Fig 2:**
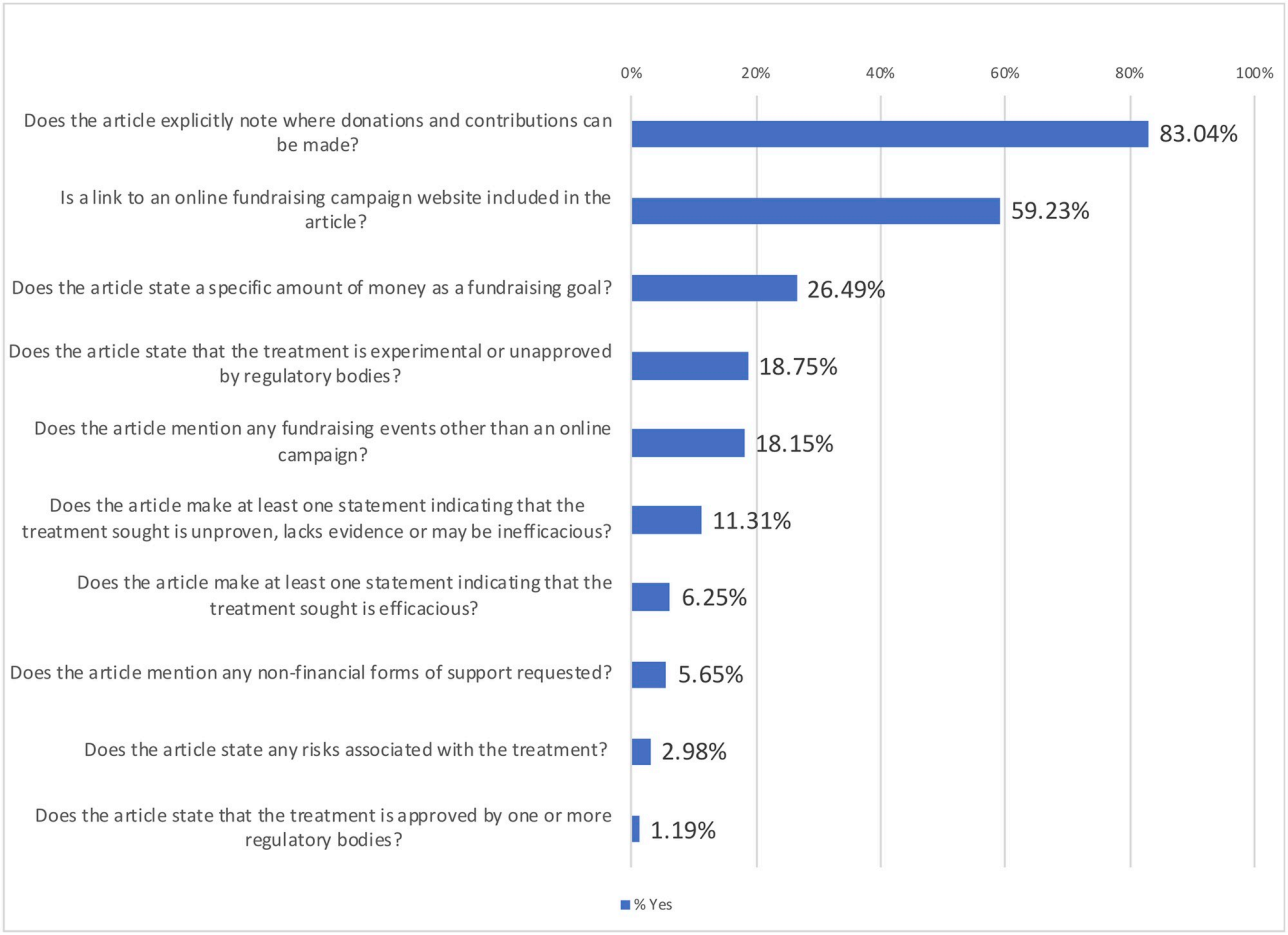
Results of multiple binary coding questions.

Fig 2 summarizes the results of several coding categories, which were focused on establishing the presence or absence of certain types of statements or information. Notably, 11.31% of articles made a statement indicating that the treatment sought is unproven, lacks evidence or may be inefficacious, and 18.75% stated that the treatment is experimental or unapproved by regulatory bodies. Together, 70 (20.83%) of the articles made at least one of these statements.

Most articles (83.04%) noted where donations and contributions can be made, and 59.23% actually included a hyperlink to an online crowdfunding campaign website. Of the 70 articles that indicated the treatment may be inefficacious or is experimental, 56 (80.00%) noted where contributions could be made and 36 (51.43%) hyperlinked directly to an online crowdfunding campaign. Over a quarter (26.49%) of the articles mentioned a specific monetary goal for the fundraising campaign.

In several of the articles in which unproven therapies were being sought, there was no mention of the possibility of inefficacy or the experimental nature of the intervention. These articles often noted where contributions could be made and/or linked directly to them (see Box 1).

**Box 1. Examples of questionable/unproven treatments sought via crowdfunding campaigns, as mentioned in articles where no statements were made indicating treatment was potentially inefficacious or experimental**

1. “Preston has turned to alternative treatments instead of chemotherapy and radiation to prolong his quality of life, she said. He’s done extensive research and feels confident in them, she added. He’s currently receiving reiki, a Japanese form of energy healing.” (Saskatoon Star Phoenix, 2016-04-04, links to campaign, brain cancer)
2. “Her only chance of survival is treatment in Florida, what doctors call chemo on steroids that kills the Lyme and rebuilds her system. Jimmelynn’s husband, Rodney, now administers what treatments he can here at home between visits to Florida because their insurance doesn’t cover the costs.” (Fox 59, 2017-08-10, links to campaign, Lyme disease)
3. “Now she wants to visit a specialist clinic in Germany for treatment as she believes a cocktail of vitamins will help heal her and improve her quality of life.” (Daily Mail, 2017-07-17, links to campaign, Lyme disease)

The data were similar for newspapers with large readerships in both Canada and the United States. There were 100 articles from the Canadian source list and 236 from the United States list. There were only a few relatively small differences between the two jurisdictions. A greater percentage of articles with Canadian readership portrayed crowdfunding campaigns positively (53.00% positive, 4.00% negative, 39.00% neutral) than those with United States readership (39.83% positive, 4.66% negative, 51.69% neutral). A greater percentage of Canadian-read articles related to crowdfunding for cancer (63.00%) than stories read in the United States (43.22%), and a smaller percentage concerned rare disease (27.00% to 46.19%, respectively). Moreover, a greater percentage of the stories read in the United States (62.29% to 52.00%) hyperlinked to a crowdfunding website.

## Discussion

In our data set, the crowdfunding phenomenon was largely portrayed neutrally and the associated discussion was usually secondary to the common focus on individuals suffering from significant medical conditions. Generally, there was little critique or analysis of the phenomenon of crowdfunding and very little negative press. When crowdfunding campaigns were covered in more depth they were largely portrayed in a positive manner. Overall, crowdfunding was often either implicitly or explicitly endorsed.

The lack of cautionary coverage of medical crowdfunding contrasts sharply with its coverage in the academic literature, which includes more discussion of various ethical concerns and inequities.[8,9,12,14,17,25] This consistently positive media portrayal may impact the public perception of the risks and benefits of crowdfunding and, for better or worse, further legitimize it as a source of funding for medical care.

The majority of the articles mentioned where donations could be made and hyperlinked to online crowdfunding pages, highlighting how media coverage may contribute to the often mentioned equity issues.[36] While further research is needed to explore causal connections, it seems likely that media coverage would increase donations, exacerbating existing inequities created by medical crowdfunding through the “popularity contest” dynamic.[8,25] Moreover, excessive media coverage can cause problems such litigation over funds or unwanted fame.[20]

More than a fifth of the relevant articles made a statement suggesting that the treatment may be inefficacious, experimental or unapproved by regulatory bodies. The majority of these articles still noted where contributions could be made and hyperlinked to the online crowdfunding campaigns. Such reporting could be interpreted as promoting and raising money for unproven or even fraudulent therapies. While it was beyond the scope of the study to individually assess the scientific and regulatory status of the interventions sought, we did note that at least some of these articles spoke of stem cell treatments for which there is little or no supporting evidence (as noted by both the research community [37,38] and entities like the International Society of Stem Cell Research).[39,40] This is to be expected, as recent research has shown that there are a significant number of crowdfunding campaigns for unproven stem cell therapies–some of which parrot the questionable marketing claims of stem cell clinics.[15] As per Text Box 1, there were also examples of articles mentioning campaigns for unproven therapies without noting their unproven or experimental nature. Such promotion is a serious problem, as it likely helps to legitimize unproven therapies, raises funds for approaches that may harm or defraud patients, and furthers the marketing campaigns of practitioners offering them. Recent research has found that the marketing language of clinics offering unproven therapies is often copied or paraphrased directly onto campaign donation websites,[15,17] increasing the opportunity for members of the public to be exposed to false or misleading representations of science. While we appreciate that the news media is a profit-oriented industry,[41] we hope popular publications will strive to present health-focused crowdfunding stories in an evidence-based manner.

This study has limitations. The study was geographically limited to Canada and the United States. Moreover, the language was restricted to English, meaning relevant French language news articles from Québec and potentially elsewhere in Canada were not analyzed. The scope of the research was limited to illness-related crowdfunding, and so it was not fully representative of newspaper representations of all biomedical crowdfunding (such as for physical trauma). Moreover, the use of the search term “cancer” impacted the representation of ailments in the data set, though it remains a broad search term that represents a multitude of conditions. Also, the data set is slightly more than a year old, and the crowdfunding industry has continued to develop since that time. Finally, it is possible other illness-related crowdfunding articles could have existed outside the scope of the search or could have existed in less popular newspapers that fell outside the purview of this study.

Future research in this area should investigate the extent to which popular articles on crowdfunding campaigns impact the quantity of funds raised. Such data could be analyzed in many ways, including by considering or weighting the popularity of the publication in which direct links to campaign websites were found. A second layer of research could assess the pervasiveness of funding for unproven therapies across a wide range of medical conditions, expanding on this research and other work.[15,16] The stage is set for these further investigations, which should help institutions and regulators make informed decisions on how to deal with emerging crowdfunding issues.

## Supporting information

S1 Text Supplementary materials. Intercoder Reliability Results and Coding Frame.
(DOCX)

S1 Dataset Base data. (XLSX)
