# Supplementary material for: Media portrayal of illness-related medical crowdfunding: A content analysis of newspaper articles in the United States and Canada
Source: PLoS One. 2019 Apr 23;14(4):e0215805. doi: 10.1371/journal.pone.0215805 (PMC6478318; doi:10.1371/journal.pone.0215805)
Supplement: S1 Text — Intercoder Reliability Results and Coding Frame. (DOCX) [file pone.0215805.s001.docx]

**Supplementary Materials**

| **Coding Frame: Results of Intercoder Reliability Testing (n=55)** |  |  |
| --- | --- | --- |
| **Question #** | **% Agreement** |  |
| What is the main topic of this article? | 98.18% |  |
| How is the phenomenon of crowdfunding portrayed? | 100.00% |  |
| If the article discusses a specific crowdfunding campaign, how is the campaign portrayed? | 78.38% |  |
| If the article discusses ethical issues with the health-related crowdfunding phenomenon, what issues does it mention? Select all that apply. | 100.00% |  |
| If the article covers a specific patient or specific patients, who initiated the crowdfunding campaign? (choose all that apply) | 89.19% |  |
| If a specific crowdfunding campaign is covered, what ailment(s) is/are mentioned to have precipitated the need for a crowdfunding campaign? | 94.59% | **Cohen’s Kappa** |
| Does the article make at least one statement indicating that the treatment sought is efficacious? | 97.30% | .8425 |
| Does the article make at least one statement indicating that the treatment sought is unproven, lacks evidence or may be inefficacious? | 94.59% | .7702 |
| Does the article state that the treatment is approved by one or more regulatory bodies? | 100.00% | N/A (division by 0)* |
| Does the article state that the treatment is experimental or unapproved by regulatory bodies? | 97.30% | .8737 |
| Does the article state any risks associated with the treatment? | 97.30% | 0^ |
| Does the article explicitly note where donations and contributions can be made? | 94.59% | .8011 |
| Is a link to an online fundraising campaign website included in the article? | 100.00% | 1 |
| Does the article mention any fundraising events other than an online campaign? | 91.89% | .8474 |
| Does the article state a specific amount of money as a fundraising goal? | 94.59% | .8902 |
| Does the article mention any non-financial forms of support requested? | 100.00% | 1 |

* Kappa could not be computed as application of the formula resulted in a division by 0. However, there was 100% agreement, as all answers were “No”.

^ 97.30% agreement, but the only disagreement (1/37) was the sole “Yes” response. This score was thus created, showing limitations of Kappa analysis where binary coding responses have highly disparate frequency.

**Coding Framework**

1. URL
2. Date of Publication
3. News Source
4. Article Title
5. What is the main topic of this article?
   1. The health-related crowdfunding phenomenon
   2. Business news relating to a crowdfunding company/platform
   3. A patient in need of treatment or funds who is using crowdfunding
   4. A patient in need of treatment or funds, where crowdfunding is mentioned but is not currently being used by the patient
   5. Other
   6. Not an article related to crowdfunding
6. How is the phenomenon of crowdfunding portrayed?
   1. In a supportive or positive manner
   2. In a cautionary or negative manner
   3. With a neutral characterization
7. If the article discusses a specific crowdfunding campaign, how is the campaign portrayed?
   1. In a positive or supportive manner
   2. In a negative or unsupportive manner
   3. In a neutral manner
   4. Other
   5. Does not cover a specific crowdfunding campaign
8. If the article discusses ethical issues with the health-related crowdfunding phenomenon, what issues does it mention? Select all that apply.
   1. Crowdfunding for unproven therapies
   2. Privacy concerns
   3. Concerns over possible misuse of funds by campaign creator/manager or funding recipient
   4. Concern about equitable distribution of funds to those in need (i.e. donating to individuals instead of charity)
   5. Concern with fees and/or profits made by crowdfunding platforms
   6. Other
   7. Ethical issues with health-related crowdfunding were not discussed
9. If the article covers a specific patient or specific patients, who initiated the crowdfunding campaign? (choose all that apply)
   1. Patient
   2. Family
   3. Extended Family
   4. Friend(s)
   5. Stranger(s)
   6. Other
   7. No mention of who started the campaign
   8. No mention of specific campaign
10. If a specific crowdfunding campaign is covered, what ailment(s) is/are mentioned to have precipitated the need for a crowdfunding campaign?
    1. Cancer
    2. Rare Disease (as stated by the article)
    3. Organ Failure
    4. Other
    5. A specific crowdfunding campaign is not covered

**YES/NO**

1. Does the article make at least one statement indicating that the treatment sought is efficacious?
2. Does the article make at least one statement indicating that the treatment sought is unproven, lacks evidence or may be inefficacious?
3. Does the article state that the treatment is approved by one or more regulatory bodies?
4. Does the article state that the treatment is experimental or unapproved by regulatory bodies?
5. Does the article state any risks associated with the treatment?
6. Does the article state any risks associated with failure to get treatment?
7. Does the article explicitly note where donations and contributions can be made?
8. Is a link to an online fundraising campaign website included in the article?
9. Does the article mention any fundraising events other than an online campaign?
10. Does the article state a specific amount of money as a fundraising goal?
11. Does the article mention any non-financial forms of support requested?
12. If the article covers a specific patient, is the treatment enabled by the crowdfunding complete at the time of publication?
